# Clinical implications of immune checkpoint inhibitor in chemotherapy-resistant advanced endometrial cancer with a pathogenic *POLE* mutation: a case report

**DOI:** 10.1007/s13691-026-00879-x

**Published:** 2026-06-29

**Authors:** Mina Oi, Akitoshi Yamamura, Tetsuya Ishibashi, Teruki Yoshida, Sayuri Takahashi, Shota Kanbayashi, Hirohiko Tani, Kenzo Kosaka, Masayo Ukita

**Affiliations:** Department of Obstetrics and Gynecology, Shizuoka General Hospital, 4-27-1 Kita Ando, Aoi-ku, Shizuoka, Shizuoka City, 420-8527 Shizuoka Japan

**Keywords:** *POLE*, Endometrial cancer, Immune checkpoint inhibitor, Immune-related Adverse Events, Comprehensive genomic profiling

## Abstract

The *POLE*-ultramutated subtype of endometrial cancer is associated with a favorable prognosis and is most frequently presents at an early stage. Even in advanced disease, which is uncommon, available data suggest favorable outcomes and a potentially limited incremental benefit of immunotherapy. We report a case of chemotherapy-resistant, *POLE*-mutated advanced endometrial cancer that showed a remarkable and durable response to immunotherapy. A 54-year-old woman was diagnosed with stage IVB endometrial cancer (endometrioid carcinoma, grade 3). Despite first-line therapy consisting of paclitaxel plus carboplatin and cytoreductive surgery, the disease progressed. Subsequent doxorubicin plus cisplatin chemotherapy also failed to achieve an objective response. Comprehensive genomic profiling revealed a pathogenic *POLE* mutation (p.V411L) with a markedly elevated tumor mutational burden of 174 mutations/Mb, while the tumor was microsatellite stable. Pembrolizumab was initiated, resulting in substantial regression of peritoneal metastases and multiple lymph node metastases. Although treatment was interrupted because of immune-related adverse events, disease has remained controlled for more than 2 years after discontinuation. This clinical course suggests that timely comprehensive genomic profiling may provide clinically actionable molecular information to guide treatment modification, even in advanced *POLE*-mutated endometrial cancer showing chemotherapy resistance. It also highlights that immunotherapy may play an important role in achieving durable disease control in this molecularly defined subset.

## Introduction

Endometrial cancer has traditionally been classified into two types—estrogen-dependent (type I) and estrogen-independent (type II). However, this binary classification does not fully account for the marked heterogeneity in clinical outcomes. In 2013, The Cancer Genome Atlas (TCGA) analyzed comprehensive genomic profiling of 373 endometrial cancers and proposed four molecular subgroups defined by characteristic genomic alterations: *POLE* (ultramutated), microsatellite instability (MSI; hypermutated), copy-number low (endometrioid), and copy-number high (serous-like), each showing distinct prognostic associations [1]. Subsequently, the Proactive Molecular Risk Classifier for Endometrial Cancer (ProMisE) was developed as a clinically applicable classifier incorporating immunohistochemistry and *POLE* sequencing. In the 5th edition of the World Health Organization (WHO) classification published in 2020, a molecular classification system was formally adopted. In this framework, endometrioid carcinoma is categorized into four molecular subtypes: *POLE*-ultramutated, mismatch repair-deficient (dMMR), p53-mutant, and no specific molecular profile (NSMP) [2]. Moreover, the updated 2023 International Federation of Gynecology and Obstetrics (FIGO) staging system integrated molecular classification into staging, reinforcing its role as a key determinant of treatment stratification and prognostic assessment [3]. Among these, the *POLE*-ultramutated subtype accounts for approximately 7–10% of endometrial cancers. It is characterized by an exceptionally high tumor mutational burden (TMB) and is associated with an excellent prognosis; 5-year survival rates of 98–100% have been reported [4, 5]. Notably, 89.5% of *POLE*-ultramutated tumors are diagnosed at stage I–II [4], and because identification requires DNA sequencing, reports of advanced-stage disease with confirmed *POLE* mutations remain uncommon.

In systemic therapy for advanced endometrial cancer, treatment selection based on molecular classification has not yet been fully established. In *POLE*-ultramutated endometrial cancer, treatment de-escalation—such as omission of adjuvant therapy—has mainly been investigated in high-risk group [6]. By contrast, because *POLE*-ultramutated tumors exhibit an exceptionally high TMB and are considered highly immunogenic, the clinical significance and optimal role of immunotherapy—particularly in advanced or chemotherapy-resistant settings—remain unclear, and no definitive consensus has been reached.

We report a case of stage IVB endometrial cancer harboring a pathogenic *POLE* mutation, in which chemotherapy failed to achieve an objective response, whereas treatment with an immune checkpoint inhibitor (ICI) resulted in a marked and durable response. This case suggests that, even in *POLE*-ultramutated endometrial cancer—generally regarded as a favorable-prognosis subtype—immunotherapy may represent an important therapeutic option in advanced or chemotherapy-resistant settings. We present this case and discuss its potential clinical implications. The timely use of comprehensive genomic profiling (CGP), when chemotherapy resistance was suspected, enabled the identification of a pathogenic *POLE* mutation and provided an important biological rationale for reconsidering an immunotherapy-based treatment strategy. Furthermore, this case illustrates that molecular genetic information obtained at an appropriate clinical time point can support treatment decision-making in advanced or chemotherapy-resistant *POLE*-mutated endometrial cancer.

## Case report

A 54-year-old nulligravid woman presented at a local clinic with a 6-month history of abnormal uterine bleeding, which was recently accompanied by lower abdominal pain. On pelvic examination, the uterus was markedly enlarged, forming a fixed pelvic mass with the adnexa. Transvaginal ultrasonography demonstrated a mass filling the uterine cavity, along with bilateral ovarian tumors containing solid components. She was referred to our institution for further evaluation and treatment. Serum cancer antigen (CA)125 and CA19-9 levels were 547 U/mL and 370 U/mL, respectively. Endometrial biopsy showed endometrioid adenocarcinoma, grade 2, with extensive necrosis and dedifferentiated components.

**Fig. 1:**
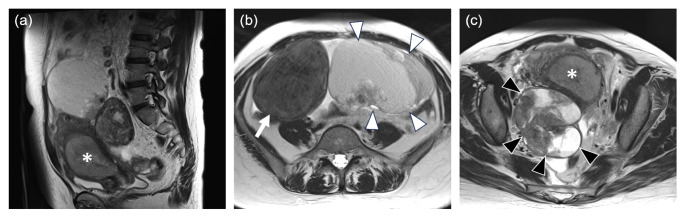
Pelvic MRI at Initial Presentation (T2-Weighted Images). (**a**) A mass (*) filled the uterine cavity with > 50% myometrial invasion.(**b**) The left ovarian tumor (white arrowhead) measured approximately 12 cm in maximal diameter and contained mixed cystic and solid components. A uterine leiomyoma (arrow) was also noted on the left side of the uterine fundus. (**c**) The right ovarian tumor (black arrowhead) measured approximately 12 cm in maximal diameter and contained mixed cystic and solid components. The intrauterine mass (*) was also visible on the same slice. MRI, magnetic resonance imaging

Contrast-enhanced pelvic magnetic resonance imaging (MRI) and computed tomography (CT) of the chest, abdomen, and pelvis demonstrated a large mass filling the uterine cavity with deep myometrial invasion (> 50%). Bilateral ovarian tumors, each approximately 12 cm in diameter, were identified, with mixed solid and cystic components (Fig. 1). Multiple enlarged lymph nodes were identified, including the left obturator, para-aortic, bilateral cardiophrenic, internal mammary, and left supraclavicular nodes. In addition, omental fat stranding, peritoneal thickening, and a moderate volume of ascites were observed, findings consistent with peritoneal metastases and widespread nodal involvement.

Based on the imaging findings, the differential diagnosis included synchronous primary endometrial and ovarian cancers or endometrial cancer with ovarian metastases and peritoneal spread; therefore, diagnostic laparoscopy was performed. Biopsy specimens obtained from peritoneal implants demonstrated histopathological features similar to those of the endometrial biopsy, including areas with dedifferentiated morphology, supporting a single diagnosis of endometrial cancer with ovarian metastases and peritoneal dissemination. Accordingly, the patient was diagnosed with endometrial cancer, staged as cT3aN2M1 (UICC 8th edition) and as stage IVB (FIGO 2008).

**Fig. 2:**
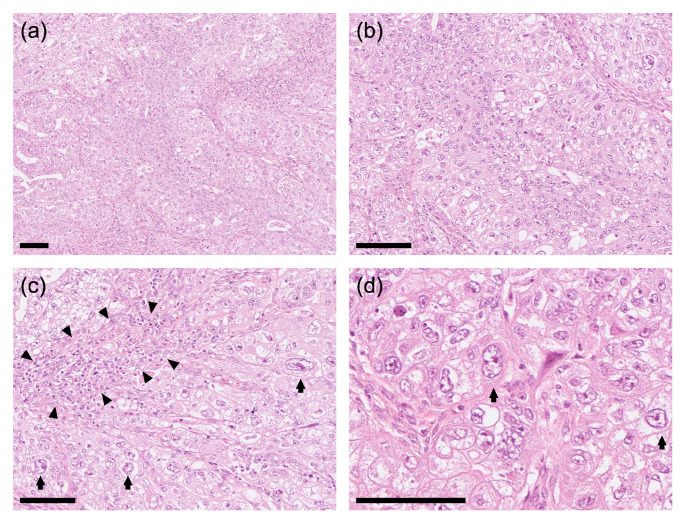
Histopathological features of the surgical specimen (**a**) Endometrioid carcinoma, grade 3, showing a predominantly solid growth pattern. (**b**) Endometrioid carcinoma, grade 3, with marked nuclear atypia in the solid tumor component. (**c**) Area rich in tumor-infiltrating lymphocytes (arrowheads) and tumor giant cells (arrow). (**d**) High-power view of tumor giant cells. Scale bar, 100 μm in all panels

First-line chemotherapy with paclitaxel plus carboplatin (TC regimen) was initiated. After two cycles, she presented to the emergency department with abdominal pain, vomiting, and profound fatigue. Contrast-enhanced CT demonstrated rupture of the left ovarian mass, and she underwent emergent surgery. Intraoperatively, the uterus and bilateral adnexa formed a fixed mass, and numerous pelvic peritoneal implants up to 2 cm in diameter were observed, some of which invaded the rectum. The left adnexal tumor was multiloculated, with a focal defect consistent with rupture. The total volume of intraperitoneal tumor spillage and ascites was 6,250 mL. She underwent total abdominal hysterectomy, bilateral salpingo-oophorectomy, pelvic peritoneal stripping, low anterior resection of the rectum, and partial omentectomy. The operative time was 9 h 36 min, and estimated blood loss was 4,310 mL. Cytoreduction was attempted; however, lymphadenectomy was not performed. Histopathological examination of the surgical specimens revealed predominantly solid growth of markedly atypical tumor cells, and the final diagnosis was endometrioid carcinoma, grade 3 (Fig. 2).

Postoperatively, four cycles of TC were administered; however, disease progression was observed, with worsening peritoneal metastases and multiple lymph node metastases. Second-line treatment with doxorubicin plus cisplatin (AP regimen) was initiated, and CGP(FoundationOne^®^ CDx) was performed concurrently. The CGP results are summarized in Table 1. A total of 24 cancer-related genomic alterations, including a pathogenic *POLE* mutation (p.V411L), were identified, and the TMB was markedly elevated at 174 mutations/Mb. Microsatellite status was assessed as microsatellite stable (MSS).

**Table 1:** Results of Comprehensive Genomic Profiling

Tumor Mutational Burden		174 Mutations/Mb	
Microsatellite Status		MS-Stable	
Gene Alterations		24 mutations	
BRCA2[p.E2301*]	POLE[p.V411L]	ATM[p.R250*]	RAD21[p.R65*]
MSH6[p.E847*]	PTEN[p.G36E]	CDK12[p.E431*]	RB1[p.E54*]
MAP2K1[p.D67N]	PTEN[p.P246L]	FBXW7[p.R658*]	RB1[p.E137*]
PIK3CA[p.R88Q]	SMARCA4[pE861K]	MAP3K13[p.R585*]	SETD2[p.R2024*]
PIK3R1[p.R348*]	TP53[p.F270C]	NF1[p.R2450*]	SETD2[p.E581*]
APC[p.R1450*]	APC[p.R2204*]	PIK3R1[p.R461*]	SMAD4[p.E520*]
Variants of Unknown Significance		151 variants	

**Fig. 3:**
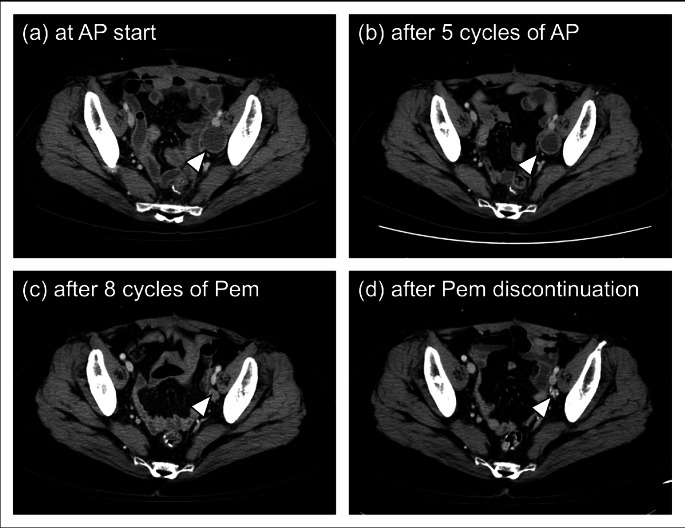
Serial CT images showing changes in the left obturator node **(a)** at AP start: The left obturator node was enlarged (white arrowhead). **(b**) after 5 cycles of AP: After five cycles of AP, the left obturator node (white arrowhead) showed minimal change in size, and the response was assessed as stable disease (SD). **(c)** after 8 cycles of Pem: After eight cycles of pembrolizumab, the left obturator node (white arrowhead) had markedly decreased in size. **(d)** after Pem discontinuation: The left obturator node (white arrowhead) remained decreased in size after discontinuation of pembrolizumab. CT, computed tomography; AP, doxorubicin/cisplatin; SD, stable disease; Pem, pembrolizumab

**Fig. 4:**
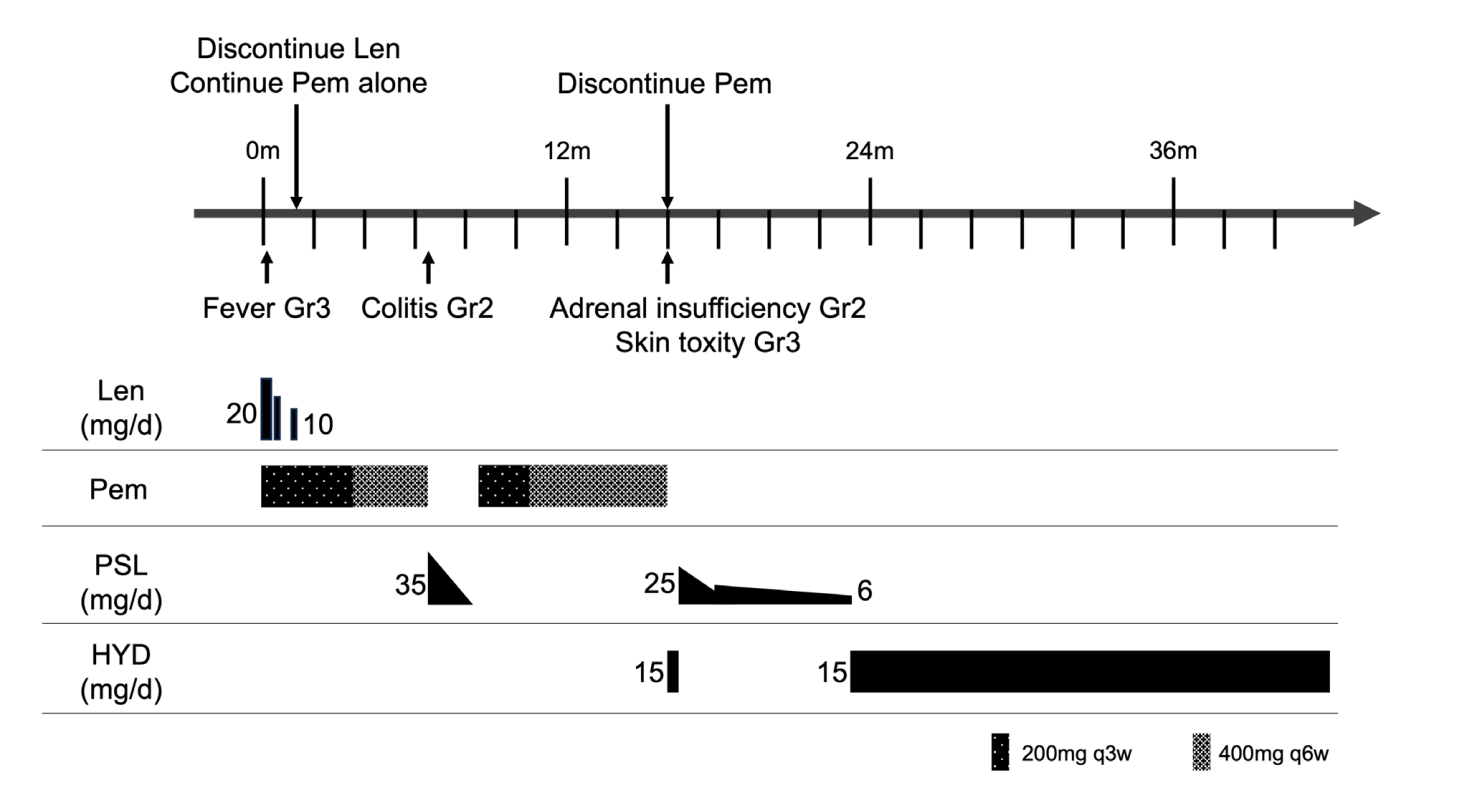
Treatment course after initiation of LP Therapy. Grade 3 fever developed during the first cycle of LP, and continued Len was considered infeasible. Treatment was therefore continued with Pem monotherapy. Immune-related colitis and adrenal insufficiency were manageable with temporary interruption of Pem and corticosteroid therapy; however, immune-related skin toxicity was severe, and Pem was permanently discontinued. LP, lenvatinib/pembrolizumab; Len, lenvatinib; Pem, pembrolizumab; PSL, prednisolone; HYD, hydrocortisone; irAE, immune-related adverse events

Five cycles of AP were administered; however, the best overall response was stable disease according to RECIST version 1.1, and treatment was discontinued due to treatment-related diarrhea (Fig. 3a, b). The regimen was subsequently switched to lenvatinib plus pembrolizumab (LP). The clinical course after initiation of LP is shown in Fig. 4. Ten days after the first cycle, the patient developed a fever exceeding 38 °C, and lenvatinib-related fever was suspected; it was therefore withheld. After rechallenge, grade 3 fever recurred, and continuation of lenvatinib was deemed infeasible. Given the high TMB, pembrolizumab monotherapy was continued. Following initiation of pembrolizumab, peritoneal metastases and lymph node metastases regressed, and tumor markers normalized after five cycles.

After seven cycles of pembrolizumab, the patient developed grade 2 diarrhea. Pembrolizumab was withheld because immune-related colitis was suspected, and the symptoms promptly improved with prednisolone at 35 mg/day. Tumor lesions continued to regress during the treatment interruption, and pembrolizumab was resumed after a 2-month treatment interruption following tapering of prednisolone. After 16 cycles of pembrolizumab, grade 2 adrenal insufficiency developed, and hydrocortisone (15 mg/day) was initiated. Around the same time, the patient developed grade 3 skin toxicity; a biopsy demonstrated subepidermal blistering dermatitis, consistent with an immune-related dermatologic adverse event. Pembrolizumab was discontinued again, and systemic corticosteroid therapy was administered. Because the skin toxicity recurred during steroid tapering, the dermatology team recommended against resumption with pembrolizumab in the setting of ongoing disease control. On CT performed 3 months after pembrolizumab discontinuation, tumor lesions had further regressed and remained controlled; a durable response has been maintained for 2 years after discontinuation of pembrolizumab (Fig. 3c, d).

## Discussion

*POLE* encodes the catalytic subunit of DNA polymerase ε (Pol ε), a key replicative polymerase that plays a central role in maintaining high-fidelity DNA replication in eukaryotic cells. Pol ε primarily synthesizes the leading strand and preserves replication fidelity through its intrinsic 3′→5′ exonuclease proofreading activity, thereby contributing fundamentally to genome stability [7, 8].

*POLE* exonuclease domain mutations (EDMs) impair the intrinsic proofreading function of Pol ε and result in an ultramutated phenotype. Approximately 80% of *POLE*-ultramutated tumors exhibit a high mutational burden with a TMB exceeding 100 mutations/Mb, and activation of antitumor immune responses driven by the generation of abundant neoantigens is considered a key mechanism underlying their exceptionally favorable prognosis [9]. Consistent with this mechanism, *POLE*-mutated tumors have been reported to show increased expression of chemokines, such as CXCL9, CXCL10, CXCL13, and CCL5, as well as immune cell–related genes [10]. This may promote the recruitment of immune cells, including T cells and dendritic cells, into the tumor site, thereby forming a tumor microenvironment enriched with immune cell infiltration [10]. In this context, antitumor immune responses mainly mediated by CD8-positive cytotoxic T cells are induced, accompanied by activated T-cell responses characterized by increased expression of effector molecules such as IFN-γ, perforin, and granzyme B [11]. In the present case, the marked lymphocytic infiltration observed in Fig. 2c may be consistent with an immune-inflamed tumor microenvironment associated with pathogenic *POLE* mutation.

Among *POLE* EDMs, P286R and V411L are frequently observed hotspot mutations that have been established as pathogenic driver alterations [12]. In the present case, a V411L mutation was identified, with a TMB of 174 mutations/Mb. These hotspot mutations cluster near the exonuclease active site and markedly compromise proofreading activity. As a result, they produce a characteristic mutational spectrum dominated by C > A and T > G single-nucleotide substitutions, which differs from the MSI-associated hypermutation pattern observed in dMMR tumors [9].

In the present case, CGP identified 24 cancer-related genomic alterations, 19 of which showed a base-substitution pattern consistent with *POLE* proofreading deficiency. *POLE*-ultramutated tumors have been reported to frequently harbor co-occurring mutations in large genes involved in DNA replication/repair and chromatin regulation, such as *BRCA2*, *ARID1A*, *ATM*, and *NOTCH1*. However, many of these accompanying alterations are typically nonrecurrent, are not associated with biallelic inactivation, and do not appear to have distinct functional phenotypes; therefore, they are generally interpreted as passenger mutations arising as a consequence of the exceptionally high mutational burden [9, 13–15].

In a pan-cancer analysis of 450 tumors harboring *POLE* mutations, the most frequent co-occurring alteration was *TP53* (52%), followed by *ARID1A* (22%), *BRCA2* (21%), *KRAS* (21%), *NF1* (19%), *NOTCH3* (18%), *NOTCH1* (18%), *ATM* (18%), *PIK3CA* (17%), *SETD2* (17%), and *SMARCA4* (17%) [14]. In the present case, co-occurring mutations in *TP53*, *BRCA2*, *MSH6*, *ATM*, and *SMARCA4* were also identified, demonstrating a co-mutation profile characteristic of *POLE*-ultramutated tumors.

Although tumors harboring *TP53* mutations are typically assigned to the poor-prognosis p53-mutant subtype, this may not apply to cases exhibiting multiple molecular features, as in the present case. Tumors showing overlapping molecular classifier characteristics are referred to as “multiple-classifier” endometrial cancers and have been reported to account for approximately 3–6% of endometrial cancer cases [16]. In tumors with concomitant *POLE* and *TP53* mutations, early-stage grade 3 endometrioid histology is common, and characteristic pathological features of the *POLE*-ultramutated subtype—such as the presence of tumor giant cells and prominent tumor-infiltrating lymphocytes—have been described [5, 16]. Moreover, in the TCGA hierarchical clustering analysis of endometrial cancer based on single nucleotide variants and somatic copy number alterations, most *POLE* mut–p53abn cancers clustered with single-classifier *POLE*-mutated cancers, and survival outcomes were significantly better than those of single-classifier p53abn cancers [16]. Collectively, these findings support the appropriateness of managing *POLE* mut–p53abn cancers as belonging to the *POLE*-ultramutated subtype.

By contrast, tumors harboring both *POLE* mutations and dMMR are not biologically uniform, and their clinical behavior appears to diverge according to the pathogenicity of the *POLE* variant. In tumors with pathogenic *POLE* EDMs, as in the present case, an ultramutated phenotype is maintained even in the presence of concomitant MMR gene alterations, and clinical outcomes are comparable to those of *POLE*-ultramutated tumors. In contrast, tumors with nonpathogenic *POLE* EDMs coexisting with dMMR have been reported to show outcomes similar to those of *POLE*–wild-type tumors [9].

As noted above, the *POLE*-ultramutated subtype frequently exhibits a high TMB; however, approximately 80% of these tumors are microsatellite stable. This indicates that the ultrahigh TMB driven by *POLE* proofreading deficiency arises through a molecular mechanism that is largely independent of mismatch repair deficiency–associated MSI [1, 9]. TMB-high is considered an independent predictive biomarker for response to ICIs, regardless of microsatellite status [17]. In an analysis of 82 patients with advanced solid tumors harboring pathogenic *POLE* mutations treated with ICI monotherapy, high rates of disease control (82.4%) and objective response (47.1%) were observed irrespective of microsatellite status [17]. However, reports describing the use of ICIs in advanced, chemotherapy-resistant *POLE*-ultramutated endometrial cancer remain limited. Previously reported cases are summarized in Table 2 [18–22].

**Table 2:** Literature review of reported cases of ICI therapy for advanced or recurrent endometrial cancer with *POLE* mutations

Author (Year)	Age	Histology	Disease setting	POLE hotspot mutation	TMB	MSI status	Prior chemotherapy	Response to chemotherapy	ICI	Best response to ICI	irAE
Mehnert et al. (2016)^18^	53	Endometrioid carcinoma, G3	Recurrent	V411L	High	MSS	TAP	details not reported	Pembrolizumab	PR	Rash Liver dysfunction Fever
Santin et al. (2016)^19^	57	mixed clear cell and Endometrioid carcinoma	Recurrent	P286R	117 mut/Mb	MSS	TAP→TC→paclitaxel	PD	Nivolumab	PR	Not reported
Veneris et al. (2019)^20^	49	Endmetrioid carcinoma, G2	Recurrent	V411L	231 mut/Mb	MSS	TC	PD	Pembrolizumab	PR	Not reported
Ebner et al. (2025)^21^	31	Poorly differentiated high-grade endometrioid carcinoma	Advanced	A456P	280 mut/Mb	MSS	unused	unused	Pembrolizumab	CR	Not reported
Charewycz et al. (2025)^22^	67	Undifferenciated uterine sarcoma	Recurrent	V411L	171 mut/Mb	Not reported	unused	unused	Pembrolizumab	CR	Hypothyroidism Xerostomia
This report	54	Endometrioid carcinoma, G3	Advanced	V411L	174 mut/Mb	MSS	TC → AP	PD → SD	Pembrolizumab	PR	Colitis Adrenal insufficiency Skin toxicity

MSI, microsatellite instability; MSS, microsatellite stable; TMB, tumor mutation burden; TAP, paclitaxel/doxorubicin/cisplatin; TC, paclitaxel/carboplatin; PD, progressive disease; SD, stable disease; PR, partial response; CR, complete response; ICI, immune checkpoint inhibitor; irAE, immune-related adverse events

The *POLE*-ultramutated subtype is characterized by a highly immunogenic tumor microenvironment with abundant tumor-infiltrating lymphocytes and is considered a biologically favorable subgroup of endometrial cancer. In the ENGOT-EN6-NSGO/GOG-3031/RUBY trial enrolling patients with advanced or recurrent endometrial cancer, an exploratory subgroup analysis based on molecular classification was presented at ESMO 2024 [23]. In that analysis, although the number of *POLE*-ultramutated cases was extremely small, outcomes appeared excellent even with chemotherapy alone, and the incremental benefit of adding an ICI was not clearly demonstrated.

In contrast, the present case involved highly advanced stage IVB disease, and neither first-line TC nor second-line AP achieved an adequate tumor response. For advanced endometrial cancer after platinum-based chemotherapy, lenvatinib plus pembrolizumab therapy is currently regarded as a standard treatment based on the results of the phase III KEYNOTE-775 trial [24]. Although pembrolizumab monotherapy could theoretically have been effective in the present case because of the pathogenic *POLE* mutation and markedly high TMB, lenvatinib plus pembrolizumab therapy was selected in accordance with standard clinical practice. After early discontinuation of lenvatinib because of adverse events, pembrolizumab monotherapy was continued, followed by a clear clinical benefit, including normalization of tumor markers and radiographic regression of the peritoneal and nodal lesions. Although immune-related adverse events (irAEs) occurred during treatment, necessitating permanent discontinuation of pembrolizumab, a durable response has been maintained for more than 2 years after cessation. In a study of patients with non–small cell lung cancer who discontinued ICIs because of irAEs, post-discontinuation survival was reported to depend on the duration of ICI exposure. The median post-discontinuation progression-free survival (PFS) and overall survival (OS) in the overall cohort were 12.7 months and 43.7 months, respectively, whereas patients treated for more than 6 months showed markedly prolonged outcomes, with median post-discontinuation PFS and OS of 25.8 months and 86.9 months, respectively [25]. In the present case, the duration of pembrolizumab treatment was 16 months (including a 2-month interruption), and the subsequent clinical course was consistent with these observations.

These findings suggest that, although *POLE*-ultramutated endometrial cancer is generally regarded as a favorable-prognosis subtype, immunotherapy may play a pivotal role in achieving disease control in selected patients with advanced or chemotherapy-resistant disease. Importantly, this case does not contradict the RUBY trial findings. Instead, it highlights that, even in advanced *POLE*-ultramutated endometrial cancer, ICIs may represent an important therapeutic option at the individual patient level.

In Japan, CGP testing under the universal health insurance system is generally available only for patients for whom no standard treatment is available, or for whom the remaining standard treatment options are expected to become limited in the near future. Therefore, a clinical framework for incorporating genomic information into treatment selection before initial therapy has not yet been fully established. In addition, the implementation of the FIGO 2023 classification in routine clinical practice remains limited. However, clinical studies such as the UPFRONT [26] and FIRST-Dx trials [27] are currently evaluating the utility of treatment strategies based on genomic information obtained before initial therapy, suggesting that early identification of molecular characteristics and their integration into therapeutic decision-making may become increasingly important. Although CGP testing was not performed before initial treatment in the present case, timely identification of a pathogenic *POLE* mutation after the recognition of poor response to chemotherapy provided a biological rationale for considering ICI therapy and offered a new perspective for treatment selection. In this regard, the present case highlights the practical clinical relevance of precision medicine by illustrating how molecular genetic information obtained at an appropriate clinical time point can contribute to the reconsideration of treatment strategy in advanced or recurrent endometrial cancer.

In conclusion, we reported an informative case of stage IVB endometrial cancer harboring a pathogenic *POLE* mutation, in which the tumor was resistant to chemotherapy but showed a marked and durable response to ICI. Although *POLE*-ultramutated endometrial cancer is generally considered a favorable-prognosis subtype and some data have suggested that the incremental benefit of immunotherapy may be limited in advanced disease. Nevertheless, the present clinical course indicates that molecular genetic information obtained through timely CGP, when treatment resistance was suspected, can contribute to reconsideration of treatment strategy in advanced or chemotherapy-resistan*t POLE*-mutated endometrial cancer. This case also supports the potential clinical relevance of immunotherapy for achieving durable disease control in selected patients with this molecularly defined subtype. Further efforts are warranted to facilitate the implementation of *POLE* mutation analysis in routine clinical practice and to clarify the clinical significance of immunotherapy for *POLE*-mutated advanced endometrial cancer through additional case reports and further clinical investigation.

## Data Availability

No datasets were generated or analyzed during the current study beyond the information presented in this case report.
